# The impact of maternal psychopathology on psychomotor development trajectories in infants born after a threatened preterm labour from 6 to 30 months of age

**DOI:** 10.1192/j.eurpsy.2024.1692

**Published:** 2024-08-27

**Authors:** J. Andreu, J. Buesa, N. Gómez, F. Ghosn, A. Moreno, L. Campos, B. Almansa, C. Zapata, M. Lizarán, A. García-Blanco

**Affiliations:** ^1^Psychiatry, Hospital Universitario y Politécnico La Fe de Valencia; ^2^Psychology, La Fe Research Institute; ^3^Psychology, Hospital Universitario y Politécnico La Fe de Valencia, Valencia, Spain

## Abstract

**Introduction:**

Threatened preterm labor (TPL) represents an adverse prenatal event that can impact maternal mental health in the long term. Additionally, this prenatal event can disrupt fetal neurodevelopment, primarily during the third trimester of pregnancy when neuronal connections in the fetus are established. Indeed, infants born following TPL exhibit delayed communication and socio-individual skills at 6 months of age, regardless of prematurity. Furthermore, maternal mental health during the postpartum period can also influence the offspring’s psychomotor development.

**Objectives:**

The aim of this study is to examine the impact of maternal psychopathology on psychomotor development trajectories in infants born after a TPL from 6 to 30 months of age.

**Methods:**

This prospective cohort study recruited 117 mother–child pairs who suffered from a TPL. Psychomotor assessment was performed at 6 and 30 months of age using the communication and socio-individual subscales of Ages & Stages Questionnaires for psychomotor development. A regression model was carried out, including gestational age at birth, maternal anxiety trait, maternal history of psychological traumas, prenatal and postnatal maternal depression, anxiety, and cortisol as well as parenting stress as predictors.

**Results:**

Increased communication delays were associated with higher maternal anxiety levels (p < 0.001), elevated maternal depression scores (p= .0003), and increased cortisol levels (p = .004) during postpartum. Similarly, elevated cortisol levels after 6 months postpartum were predictive of increased Personal-Social delays (p = .0018).

**Conclusions:**

Maternal postpartum psychopathology was the main determinant of the course of psychomotor developmental disturbances. Therefore, infants born after TPL, whose mothers display postpartum psychopathology, should be identified and considered for psychological treatment to improve psychomotor delays in infants.

**Disclosure of Interest:**

None Declared

